# N6‐Methyladenosine Demethylase FTO Mitigates Cerebral Ischemia–Reperfusion Injury by Suppressing AQP4 Expression

**DOI:** 10.1002/cns.71011

**Published:** 2026-08-04

**Authors:** Wei Chen, Luyuan Cao, Xiaoxiao Zheng, Chenyuan Yu, Xiaoqiang Zhu, Qing Wang, Li Zheng, Jiahua Lan, Cheng Jiang, Hongwei Wang

**Affiliations:** ^1^ Provincial Key Laboratory of Precise Diagnosis and Treatment of Abdominal Infection, Department of General Surgery Sir Run Run Shaw Hospital, School of Medicine, Zhejiang University Hangzhou Zhejiang China; ^2^ School of Basic Medical Sciences and Forensic Medicine Hangzhou Medical College Hangzhou Zhejiang China; ^3^ Cancer Institute of Integrated Traditional Chinese and Western Medicine, Key Laboratory of Cancer Prevention and Therapy Combining Traditional Chinese and Western Medicine of Zhejiang Province Zhejiang Academy of Traditional Chinese Medicine, Tongde Hospital of Zhejiang Province Hangzhou Zhejiang China; ^4^ Department of Anesthesiology Tongde Hospital of Zhejiang Province, Zhejiang Academy of Traditional Chinese Medicine Hangzhou Zhejiang China

**Keywords:** AQP4, cerebral ischemia–reperfusion injury, FTO, *Hspa1a*, m6A modification

## Abstract

**Aim:**

In patients with acute ischemic stroke, reperfusion therapy can compromise the blood–brain barrier, resulting in secondary cerebral ischemia–reperfusion injury (CIRI). This study investigated the involvement of the N6‐methyladenosine (m6A) demethylase FTO in the pathogenesis of CIRI and explored the underlying molecular mechanisms.

**Methods:**

We established a middle cerebral artery occlusion (MCAO) mouse model in vivo and an oxygen–glucose deprivation/reperfusion (OGD/R) model in primary astrocytes in vitro.

**Results:**

FTO expression was significantly downregulated in both the in vivo and in vitro CIRI models. Upregulation of FTO via plasmid transfection significantly attenuated cellular damage and enhanced astrocyte viability. At the molecular level, m6A‐modified transcripts were enriched in the MAPK signaling pathway during cerebral ischemia–reperfusion. Notably, Heat Shock Protein Family A (Hsp70) Member 1A (*Hspa1a*) was identified as a key target. The reduction in FTO expression led to increased m6A methylation levels on *Hspa1a* mRNA, thereby promoting its degradation. Furthermore, the cellular damage induced by FTO silencing was alleviated by *Hspa1a* overexpression or the silencing of Aquaporin‐4 (AQP4).

**Conclusions:**

This study demonstrates that FTO mitigates CIRI by stabilizing *Hspa1a* mRNA, which in turn suppresses AQP4 expression. Consequently, FTO represents a promising therapeutic target for the treatment of ischemic stroke.

## Graphical abstract

Mechanism of FTO regulating *Hspa1a* m6A modification for protection against CIRI. A CIRI leads to a decrease in FTO expression in astrocytes followed by an increase in *Hspa1a* m6A modification, which promotes HSPA1A degradation. Simultaneously, the NF‐κB pathway becomes activated, and AQP4 expression is upregulated. These consequences aggravate cell injury and apoptosis further.

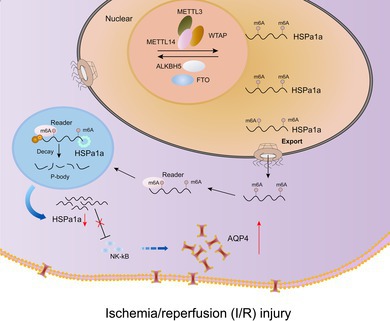

## 1 Introduction

Acute ischemic stroke (AIS) is a leading cause of long‐term disability and death worldwide [1]. Currently, reperfusion therapy and cerebroprotective strategies are considered to hold great potential for augmenting the efficacy of recanalization procedures [2]. However, reperfusion can paradoxically exacerbate tissue damage by compromising the blood–brain barrier, leading to cerebral ischemia–reperfusion injury (CIRI). This secondary injury increases neuronal cell death via apoptosis or necrosis and expands the cerebral infarct volume [3, 4, 5]. Furthermore, CIRI contributes to high rates of disability, recurrence, and mortality, placing a significant burden on patients, families, and society [6, 7]. Consequently, there is an urgent need to investigate the mechanisms underlying CIRI and to identify novel preventive or therapeutic targets to guide drug development and clinical treatment.

N6‐methyladenosine (m6A) methylation, the most prevalent post‐transcriptional modification of RNA [8], regulates critical cellular processes such as oxidative stress, apoptosis, and autophagy [9, 10, 11]. Studies utilizing methylated RNA immunoprecipitation sequencing (MeRIP‐seq) and RNA‐Seq analysis have revealed significant alterations in m6A modification levels, distribution, and associated gene expression profiles in cerebral tissue following ischemia–reperfusion [12]. FTO, a key m6A demethylase, has been shown to mitigate CIRI by reducing damage to gray and white matter, enhancing the recovery of motor function and cognition, and alleviating depression‐like behavior after stroke [13]. Mechanistically, FTO has been reported to demethylate NRF2 mRNA, thereby increasing NRF2 expression to suppress the oxidative stress response and attenuate CIRI‐related damage [14]. However, the precise mechanisms governing FTO‐mediated m6A modification in the context of CIRI remain to be fully elucidated.

Recent studies on post‐CIRI transcriptomes have identified numerous m6A‐modified long non‐coding RNAs (lncRNAs) and mRNAs, with significant enrichment observed in the Mitogen‐activated protein kinase (MAPK) signaling pathway [15]. Heat Shock Protein Family A (Hsp70) Member 1A (*Hspa1a*), a key gene within the MAPK pathway, functions as an endogenous protective protein. It can translocate to the nucleus to inhibit NF‐κB transcriptional activity, thereby reducing the release of proinflammatory cytokines (e.g., tumor necrosis factor α, interleukin [IL]‐1β, and IL‐6) and alleviating inflammation in CIRI [14]. Previous research has demonstrated that the absence of *Hspa1a* results in cardiomyocyte dysfunction and an impaired stress response to ischemia/reperfusion [16], whereas stabilizing *Hspa1a* mRNA enhances its expression and protective effects in myocardial ischemia–reperfusion injury [17]. These findings align with our previous observations; however, the specific contribution of m6A modification to the protective role of *Hspa1a* in CIRI remains elusive.

Emerging evidence suggests a link between FTO‐mediated m6A demethylation and *Hspa1a* in CIRI. For instance, Chokkalla et al. reported elevated m6A levels on *Hspa1a* mRNA post‐ischemia, identifying it as a target for m6A modification [18]. Given the role of FTO as a primary demethylase, its reduced activity during CIRI could drive this hypermethylation, potentially destabilizing *Hspa1a* mRNA. Conversely, the ability of FTO to stabilize mRNAs such as *NRF2* via demethylation mirrors observations in myocardial ischemia–reperfusion, where *Hspa1a* mRNA stabilization confers protection [19]. This suggests that FTO may similarly regulate *Hspa1a* in CIRI, although direct evidence is currently lacking. Therefore, the interplay between FTO, m6A modification, and the MAPK‐mediated protective effects of *Hspa1a* in CIRI warrants further exploration.

In this study, we investigated the mechanisms of FTO in CIRI using m6A modification as a focal point, employing both in vitro and in vivo models. We discovered that FTO modulates Aquaporin‐4 (AQP4) expression by destabilizing *Hspa1a* mRNA, thereby influencing repair processes in CIRI. These results provide a theoretical foundation for developing m6A‐targeted strategies for the prevention and treatment of cerebral ischemia–reperfusion injury.

## 2 Materials and Methods

### 2.1 Animals

Male C57BL/6J mice (6 weeks old; weight, 20–25 g) were purchased from GemPharmatech Co. Ltd. (Nanjing, China). The mice were housed in an environmentally controlled facility under a 12‐h light/dark cycle. Food and water were provided ad libitum. All animal experiments were conducted in accordance with the National Guide for the Care and Use of Laboratory Animals (NIH Publication No. 8023, revised 1978). The study protocols were approved by the Experimental Animal Welfare Ethics Committee of the Zhejiang Academy of Traditional Chinese Medicine.

### 2.2 Primary Astrocyte Cultivation

Mouse cerebellar astrocytes (MA‐C) were harvested from the cerebella of 7‐day‐old C57BL/6J mice, as previously described [20]. Briefly, cerebellar tissue was digested with 0.065% trypsin (Gibco, Grand Island, NY, USA) at 37°C for 15 min and centrifuged at 1500 × g for 5 min. The resulting cell pellet was treated with 0.004% DNase I (Sigma‐Aldrich, St. Louis, MO, USA) for 7 min and centrifuged again at 1500 × g for 5 min. The pellet was resuspended in tissue growth medium consisting of DMEM supplemented with 10% fetal bovine serum (FBS; Gibco). Cells were seeded at a density of 2 × 10^5^ cells/cm^2^ onto 35‐mm dishes (Corning Inc., Corning, NY, USA) pre‐coated with 100 μg/mL poly‐D‐lysine (Sigma‐Aldrich). Cultures were maintained at 37°C in a humidified atmosphere containing 5% CO_2_.

### 2.3 OGD/R Model Construction

The primary astrocyte oxygen–glucose deprivation (OGD) model was established as previously described [21]. Briefly, MA‐C were cultured in Roswell Park Memorial Institute (RPMI) 1640 medium (Gibco) under hypoxic conditions (99% N_2_ and 1% O_2_) for 24 h. Subsequently, the cells were subjected to reperfusion in RPMI‐1640 supplemented with serum (Gibco) under normoxic conditions for 24 or 48 h.

### 2.4 Cell Viability and Proliferation Assessment

MA‐C were seeded at a density of 2 × 10^4^ cells/well in 96‐well plates and cultured for 12 h, and our OGD/R model was established as described previously [22]. The cells were then exposed to CCK8 (10 μL/well), followed by incubation for 2 h and then by determination of absorbance at 450 nm. Cell proliferation was ascertained by using a Click‐iT EdU Imaging Kit (Invitrogen), according to the manufacturer's instructions.

### 2.5 Lactate Dehydrogenase (LDH) Analysis

LDH levels were determined using an LDH Cytotoxicity Assay Kit (Dojindo, Kumamoto, Japan), in accordance with the manufacturer's instructions. MA‐C cells were cultured in 6‐well plates, transfected, and subjected to the OGD/R model. Subsequently, the cells were collected, resuspended in fresh medium, and seeded into 96‐well plates. Following incubation in a CO_2_ incubator at 37°C, 20 μL of Lysis Buffer was added to the high control wells (maximum enzyme activity control), and the plates were incubated at 37°C for 30 min. Next, 100 μL of Working Solution was added to each well, and the cells were incubated at room temperature in the dark for 30 min. Finally, 50 μL of Stop Solution was added to each well, and the absorbance was measured immediately at 490 nm using a microplate reader (Manufacturer, City, Country) to determine LDH levels.

### 2.6 Cell Transfection

Small interfering RNAs (siRNAs) targeting *FTO* and *AQP4* were synthesized by GenePharma (Shanghai, China). Transfections were performed using Lipofectamine 2000 (Thermo Fisher Scientific, Waltham, MA, USA) according to the manufacturer's instructions. Additionally, FTO overexpression was induced using an adeno‐associated virus (AAV) vector, while AQP4 overexpression was achieved via plasmid transfection. The sequences of all oligonucleotides used in this study are listed in Table 1.

**TABLE 1:** Oligonucleotide sequences.

Oligonucleotide	Sequence
AQP4‐mus‐711	CCCGCAGUUAUCAUGGGAATT
AQP4‐mus‐711	UUCCCAUGAUAACUGCGGGTT
AQP4‐mus‐795	GUGGCCUUUAUGAGUAUGUTT
AQP4‐mus‐795	ACAUACUCAUAAAGGCCACTT
FTO‐Mus‐124	GCAGCUGAAAUACCCUAAATT
FTO‐Mus‐124	UUUAGGGUAUUUCAGCUGCTT
FTO‐Mus‐972	CAGGCACCUUGGAUUAUAUTT
FTO‐Mus‐972	AUAUAAUCCAAGGUGCCUGTT
FTO‐Mus‐1237	GGUGCUCCGUGAAGUUAAATT
FTO‐Mus‐1237	UUUAACUUCACGGAGCACCTT

### 2.7 Western Blotting

Cells from the different treatment groups were pooled and lysed using RIPA buffer (Invitrogen; Thermo Fisher Scientific Inc.). The protein concentration of each sample was determined using a BCA Protein Assay Kit (Sigma‐Aldrich). Subsequently, a specific quantity of protein was separated by 10% sodium dodecyl sulfate‐polyacrylamide gel electrophoresis (SDS‐PAGE) and transferred onto polyvinylidene fluoride (PVDF) membranes (Millipore, Billerica, MA, USA). The membranes were blocked with a blocking agent for 2 h at room temperature. Following blocking, the membranes were incubated overnight at 4°C with primary antibodies against FTO, HSPA1A, NF‐κB, and AQP4, followed by incubation with the corresponding secondary antibodies for 2 h at room temperature. Finally, protein bands were visualized using enhanced chemiluminescence (ECL) reagents (GE Healthcare, Piscataway, USA) and quantified using Image Lab 5.0 (Bio‐Rad, Hercules, CA, USA).

### 2.8 Reverse Transcription Quantitative Polymerase Chain Reaction (RT‐PCR)

Total RNA was extracted using TRIzol reagent (Invitrogen, Carlsbad, CA, USA) and reverse‐transcribed into complementary DNA (cDNA) using the Takara RT reagent (Takara Bio, Shiga, Japan). Subsequently, relative quantitative polymerase chain reaction (qPCR) was performed using the SYBR PrimeScript mRNA RT‐PCR Kit (Takara Bio) on an Applied Biosystems real‐time PCR system (Applied Biosystems, Foster City, CA, USA). All reactions were performed in triplicate. Relative gene expression was quantified using the comparative threshold cycle (2^−ΔΔCt^) method. The sequences of all qPCR primers are listed in Table 2.

**TABLE 2:** qPCR primer sequences.

Primer	Sequence
FTO‐Mus‐Forward	GACACTTGGCTTCCTTACCTG
FTO‐Mus‐Reverse	CTCACCACGTCCCGAAACAA
ALKBH5‐Mus‐Forward	CGCGGTCATCAACGACTACC
ALKBH5‐Mus‐Reverse	ATGGGCTTGAACTGGAACTTG
AQP4‐Mus‐Forward	ATCAGCATCGCTAAGTCCGTC
AQP4‐Mus‐Reverse	GAGGTGTGACCAGGTAGAGGA
METTL3‐Mus‐Forward	GAAACAGCTGGACTCGCTTC
METTL3‐Mus‐Reverse	GGCACGGGACTATCACTACG
METTL14‐Mus‐Forward	GCTAAGTCAAACACTCCTCCCA
METTL14‐Mus‐Reverse	TATTCTTCCAGAGGGGGCTC
YTHDF2‐Mus‐Forward	GAGCAGAGACCAAAAGGTCAAG
YTHDF2‐Mus‐Reverse	CTGTGGGCTCAAGTAAGGTTC
NF‐κB‐Mus‐Forward	ATGGCAGACGATGATCCCTAC
NF‐κB‐Mus‐Reverse	CGGAATCGAAATCCCCTCTGTT
Hspa1b‐Mus‐Forward	CCGACAAGGAGGAGTTCGTG
Hspa1b‐Mus‐Reverse	CTTGACAGTAATCGGTGCCC
Flnc‐Mus‐Forward	AACAACAGCAACTATTCGGACG
Flnc‐Mus‐Reverse	TGCGGTACATGCGCTTCTG
Dusp10‐Mus‐Forward	CCATCTCCTTTAGACGACAGGG
Dusp10‐Mus‐Reverse	GCTACCACTACCTGGGCTG
Nlk‐Mus‐Forward	TGGGCAACAACAGCCATATTT
Nlk‐Mus‐Reverse	GTGCGCCTTAACTGTAGCAG
Tnfrsf1a‐Mus‐Forward	CCGGGAGAAGAGGGATAGCTT
Tnfrsf1a‐Mus‐Reverse	TCGGACAGTCACTCACCAAGT
Gadd45b‐Mus‐Forward	CGTTCTGCTGCGACAATGAC
Gadd45b‐Mus‐Reverse	CTTCGGTTGTGCCCAATGTC
Rasgrp3‐Mus‐Forward	GGGAAAGCGGCAACACTAGAT
Rasgrp3‐Mus‐Reverse	GGGCAAGTAACTGTCGTTCAG

### 2.9 Flow Cytometry

Cell apoptosis was assessed through flow cytometry. In brief, the cells were stained using an Annexin V‐FITC Apoptosis Detection Kit (BD Biosciences, San Jose, CA, USA) according to the manufacturer's instructions. MA‐C were then cultured in six‐well plates and mixed with 100 μL of the binding buffer and 5 μL of FITC‐labeled annexin V; this was followed by incubation at 37°C in the dark for 15 min. Then, 5 μL of 50 μg/mL propidium iodide (PI) was added, followed by incubation in the dark for 5 min. Finally, 400 μL of the binding buffer was added, and within 1 h, apoptotic cells were quantified through flow cytometry.

### 2.10 Focal CIRI Model Construction

All mice were from the same batch; however, they were not assigned to the final treatment or vehicle groups prior to surgery. Instead, the procedure was as follows: all animals first underwent MCAO or sham surgery. At 24 h post‐surgery, deceased animals and those with failed modeling (infarct volume < 20% or neurological score < 5) were excluded. Only the surviving and qualified animals were then randomly assigned to experimental groups in a double‐blind manner by an investigator who was not involved in the surgical procedures or behavioral assessments. The middle cerebral artery occlusion/reperfusion (MCAO/R) model was induced as previously described [23]. Briefly, mice were anesthetized with isoflurane, and a 6–0 silicon‐coated nylon monofilament (Doccol Corporation, Redlands, CA, USA) was inserted into the left common carotid artery and advanced to occlude the origin of the middle cerebral artery (MCA) for 1 h. The filament was then withdrawn to allow reperfusion, and the incision was sutured. In the sham surgery group, the filament was inserted but not advanced into the internal carotid artery (ICA). Anesthesia was induced and maintained using inhaled isoflurane. Throughout the surgery and postoperative period, body temperature was maintained between 37.0°C and 37.5°C using heating pads and incubators [24]. Reperfusion was initiated immediately upon suture removal, and the animals were not sacrificed immediately; rather, they were maintained for designated survival periods to allow for the development of ischemic injury and subsequent behavioral assessment. Neurological function was evaluated 24 h post‐surgery using the Bederson score, a time point selected based on established protocols and supported by relevant literature [25, 26]. For the molecular analyses, mice were sacrificed at various pre‐determined intervals following the reperfusion period. At designated time points post‐surgery, mice were anesthetized with isoflurane and decapitated, and their brains were harvested. By utilizing mice with different FTO phenotypes, we established Sham control, wild‐type (MT‐MCAO/R), and FTO‐knockdown MCAO/R models. All eligible mice were randomly assigned to the various experimental groups.

### 2.11 2,3,5‐Triphenyl Tetrazolium Chloride Staining

Infarct volume was assessed using 2,3,5‐triphenyl tetrazolium chloride (TTC) staining. Brain sections were immersed in a 2% TTC solution (Manufacturer, City, Country) and incubated at 37°C in the dark for 30 min. Following staining, the sections were fixed in 10% paraformaldehyde and examined under a light microscope (Olympus, Tokyo, Japan). Infarcted tissue appeared white, while non‐infarcted tissue was stained red. The percentage of infarct volume was calculated using the formula: (infarcted area/total brain area) × 100.

### 2.12 Neurological Deficit Score Assessment

The inclusion criteria required successful model establishment, verified by a Bederson score of ≥ 1 at 24 h post‐operation. The Bederson score is a widely used standardized assessment where a score of 0 represents normal neurological function, and scores of 1–3 indicate neurological dysfunction corresponding to the severity of injury [1, 2, 3]. Consequently, mice exhibiting no neurological deficit (score = 0) or those that died during the procedure were excluded. Total animals: 96; Mortality: 16 mice died during or immediately after surgery; Exclusion: 8 mice were excluded due to a Bederson score of 0 (indicating failure of the MCAO model); and Final Sample Size: 72 mice were included in the final analysis. The 72 included mice were allocated as follows: Time‐course analysis: 36 mice were used to observe changes in m6A and m6A‐related enzymes at different time points (6 time points, *n* = 6 per time point). Neurological deficits were assessed 24 h after MCAO/R using the Bederson scoring system [25]. Briefly, mice were suspended by the tail approximately 10 cm above the examination surface. Sham control mice typically demonstrate a raised head and extend both forepaws toward the surface. Neurological function was graded on a 4‐point scale as follows: 0, no observable deficit (normal forepaw extension); 1, mild circling behavior with or without inconsistent rotation, involving fewer than 50% of attempts to rotate toward the contralateral side; 2, mild consistent circling, involving at least 50% of attempts to rotate toward the contralateral side; and 3, severe, immediate, and consistent circling, with the rotation position maintained for 1–2 s and the nose almost touching the tail. All assessments were performed by an investigator blinded to the experimental groups. A higher score indicates more severe neurological injury, with a score of 0 signifying normal function and scores of 1–3 indicating neurological impairment [24, 27, 28].

### 2.13 Hematoxylin–Eosin Staining

Twelve mice were used for histological assessment (2 groups, *n* = 6 per group). Paraffin‐embedded brain sections were deparaffinized in xylene and rehydrated through a graded ethanol series. The slides were stained with hematoxylin for 5 min, followed by eosin staining for 2 min. Finally, the sections were dehydrated, cleared, and examined under a light microscope (Olympus).

### 2.14 Immunohistochemistry

Twenty‐four mice were used for protein localization studies (4 groups, *n* = 6 per group). Paraffin‐embedded sections were deparaffinized in xylene and rehydrated through a graded ethanol series. The sections were blocked with 5% normal goat serum (Biosharp, Hefei, China) for 20 min at room temperature to reduce non‐specific binding. Subsequently, the sections were incubated overnight at 4°C with primary antibodies against FTO, NF‐κB, Hspa1a, and AQP4. Following primary antibody incubation, the sections were incubated with horseradish peroxidase (HRP)‐conjugated secondary antibodies (CST, Beverly, USA) for 30 min at room temperature. Immunoreactivity was visualized by incubation with 3,3′‐diaminobenzidine (DAB) for 10 min. Finally, the sections were counterstained with hematoxylin, dehydrated, cleared, and examined under a light microscope (Olympus).

### 2.15 Statistical Analysis

Experimental data are presented as the mean ± standard deviation (SD). All statistical analyses were performed using GraphPad Prism software (version 8.0; GraphPad Software, San Diego, CA, USA). Differences between groups were assessed using Student's *t*‐test, the Mann–Whitney *U* test, or Kruskal–Wallis test for comparisons between two groups or one‐way analysis of variance (ANOVA) followed by Tukey's post hoc test for multiple comparisons [29]. A *p*‐value of < 0.05 was considered statistically significant. We applied the Shapiro–Wilk test to assess data distribution. For datasets where normal distribution could not be confirmed or where sample sizes were limited, we transitioned to non‐parametric tests (such as the Mann–Whitney *U* test or Kruskal–Wallis test) to ensure the robustness of our conclusions.

## 3 Results

### 3.1 MCAO/R Leads to Low FTO Expression

**FIGURE 1:**
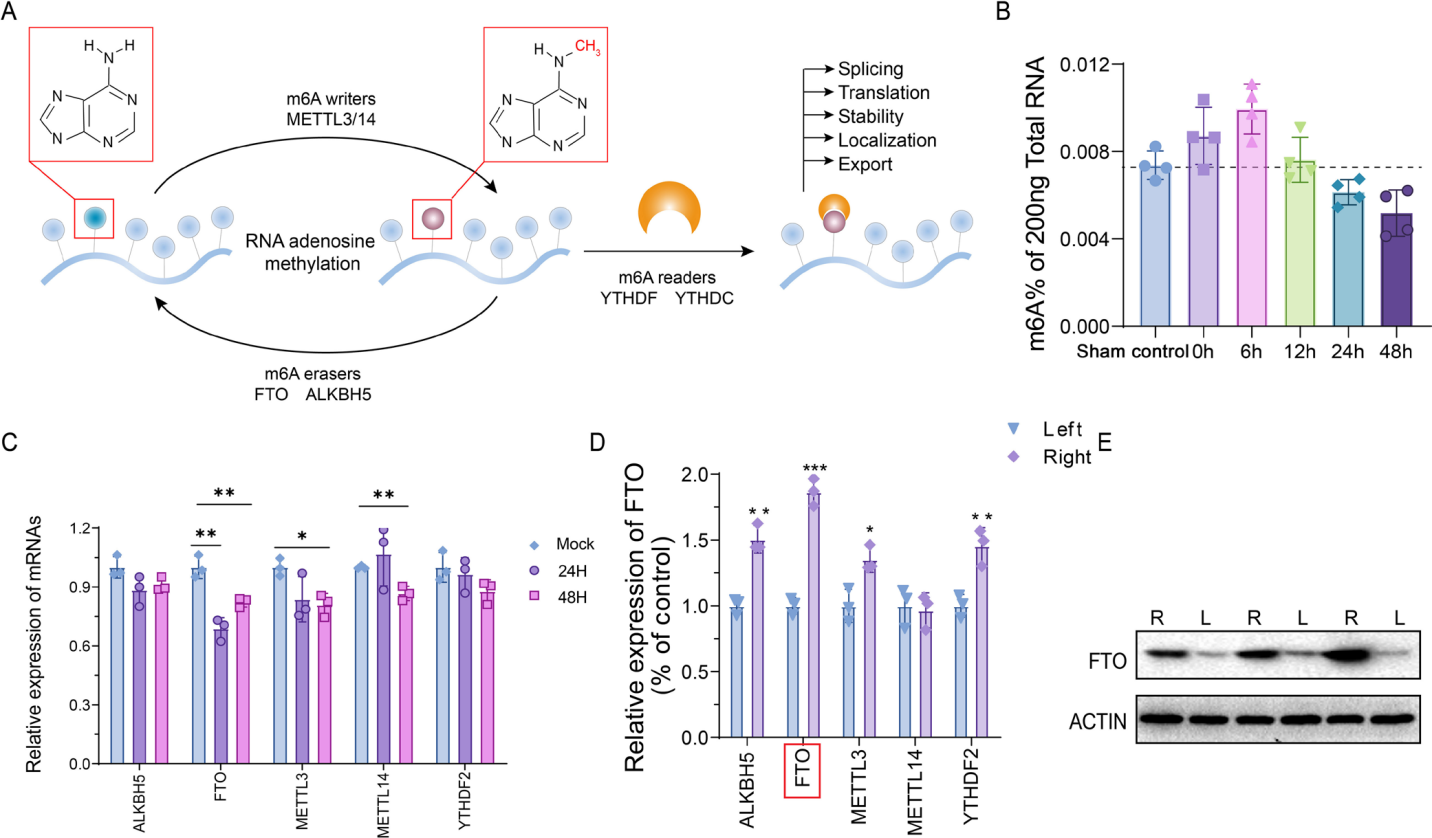
Low FTO expression in the MCAO/R and OGD/RX models. (A) Regulatory effect of m6A RNA modification on various biological processes. (B) Changes in m6A‐modified mRNA levels in mouse MCAO/R brain tissues with cerebral infarction at different time points. (C) Changes in m6A modification‐related enzyme expression in our CIRI model tissues were detected through qPCR at different time points. (D) qPCR for detecting mRNA expression in MCAO/R brain and contralateral normal brain tissue, *p < 0.05, **p < 0.01, ***p < 0.001 vs. OGD/RX. (E) Changes in FTO expression in three pairs of MCAO/R brain tissues and contralateral normal brain tissues.

The RNA m6A modification has an important role in various biological processes (Figure 1A). We assessed post‐MCAO changes in RNA m6A modification in mice with MCAO/R through qPCR and found that m6A mRNA levels increased and peaked until 6 h after cerebral infarction but then decreased (Figure 1B). Moreover, *FTO* demonstrated the highest expression, followed by *ALKBH5*, *METTL3*, *METTL14*, and *YTHDF2* (Figure 1C). We compared the qPCR results for the MCAO/R and contralateral normal brain tissues and noted that the most significant decrease in *FTO* mRNA expression (Figure 1D). Based on these findings, we hypothesized that FTO plays a crucial role in the pathogenesis of cerebral ischemia–reperfusion injury. To investigate this, we examined FTO protein expression in MCAO/R and contralateral normal brain tissues using Western blotting. The results demonstrated that the changes in FTO protein expression mirrored the alterations observed in FTO mRNA expression (Figure 1E).

### 3.2 FTO Protects Against CIRI

**FIGURE 2:**
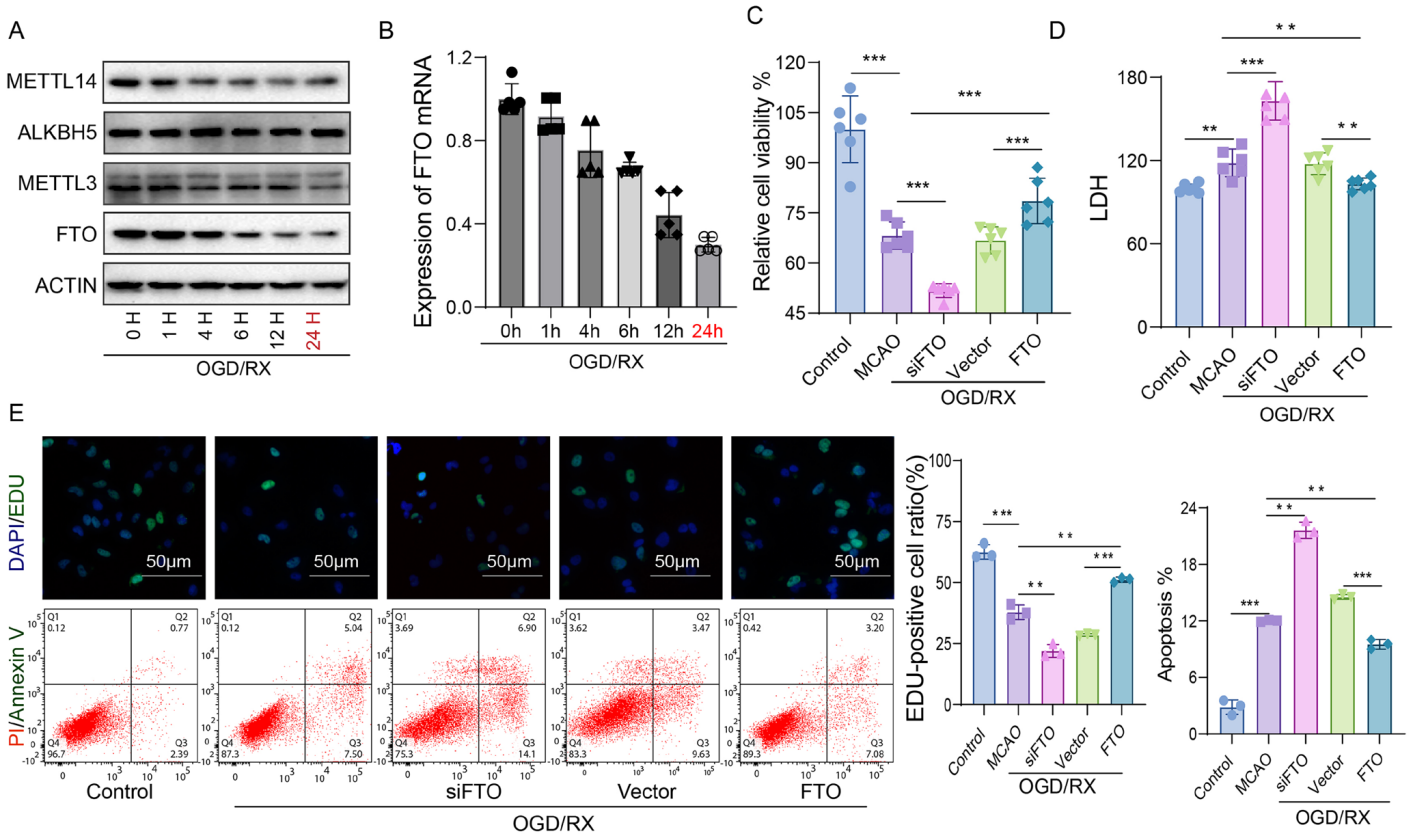
Participation of FTO in cell damage in the OGD/RX model. (A) Western blotting for alterations in m6A modification‐related enzymes FTO, ALKBH5, METTL3, METTL4, and YTHDF2 in the OGD/RX model at different time points. (B) FTO mRNA changes detected through qPCR. (C) Cell viability after transfection with FTO siRNA and FTO expression plasmid. **p < 0.01; ***p < 0.001. (D) Under OGD conditions, transfection with FTO siRNA increased LDH activity. **p < 0.01; ***p < 0.001. (E) Flow cytometry analysis demonstrating a decrease in cell apoptosis after FTO expression interference under OGD\RX. **p < 0.01; ****p < 0.001.

Next, we used Western blotting and qPCR to elucidate alterations in the expression of FTO and other m6A modification‐related enzymes over several time points in our OGD/R model. The result demonstrated that FTO expression significantly decreased over the first 24 h; moreover, with time, FTO expression decreased consistent with MCAO development (Figure 2A,B). Furthermore, we knocked down FTO expression in the MA‐C through *FTO* siRNA transfection and measured the cell viability and the levels of LDH released after OGD/R. FTO knockdown resulted in significant cell death and LDH release. However, these adverse effects were significantly attenuated following transfection with FTO‐expressing plasmids (Figure 2C,D). Similarly, our flow cytometry results demonstrated that *FTO* siRNA transfection led to a significant increase in apoptotic cell count (Figure 2E).

**FIGURE 3:**
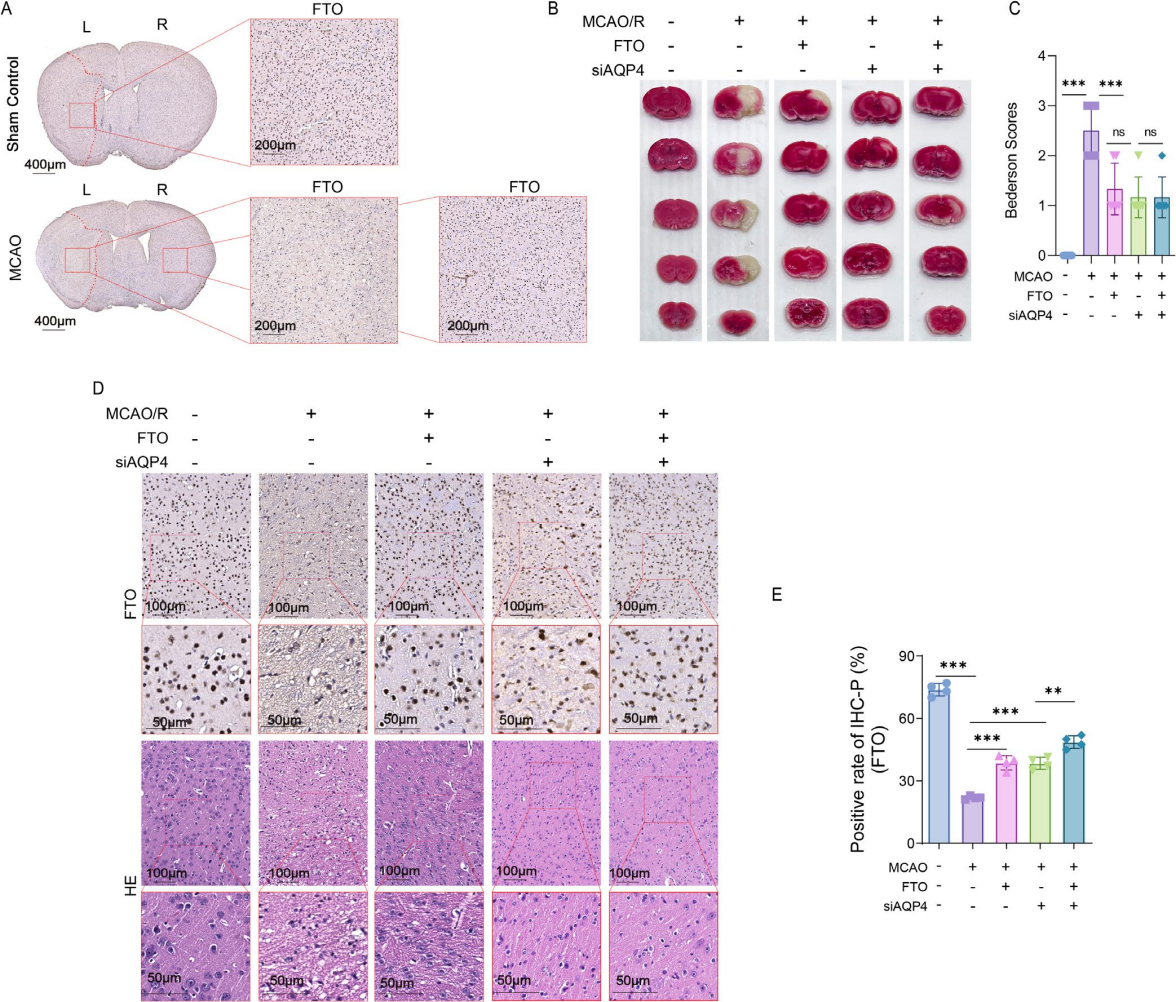
Involvement of FTO in the MCAO/R model. (A) FTO expression with and without in mouse brains after MCAO/R. The left brain is the operative side. (B) TTC staining for MCAO‐related infarct volume with or without FTO or siAPQ4 treatment. (C) Bederson scores for the neurological behavior of MCAO/R mice treated with FTO or siAQP4. (D, E) Immunohistochemistry for FTO expression in the control, MCAO/R, FTO + MCAO/R, siAPQ4 + MCAO/R, and FTO + siAPQ4 + MCAO/R groups.

To verify that FTO is involved in brain injury in our MCAO/R mice, we compared FTO expression in the left and right brain hemispheres of the MCAO/R mice with the left brain hemisphere of the Sham control mice through immunohistochemistry. The results indicated that FTO expression was significantly lower in the MCAO/R mice than in the Sham control mice (Figure 3A). Moreover, TTC staining after 24 h of reperfusion after MCAO surgery demonstrated that the infarct volume in the FTO‐overexpressing MCAO/R mice was significantly lower than that in the Sham control mice. We previously reported that AQP4 inhibition reduces CIRI‐related brain damage [22]. Therefore, when MCAO/R mice were subjected to FTO overexpression and siAQP4 treatment, the cerebral infarct volume decreased significantly; however, FTO or siAQP4 alone led to no significant cerebral infarct volume changes (Figure 3B). In addition, after 24 h of reperfusion, the Bederson score grade was significantly lower in the FTO‐overexpressing MCAO/R mice than in the untreated MCAO/R mice (Figure 3C). This result was corroborated by our immunohistochemistry and hematoxylin–eosin staining results (Figure 3D,E).

### 3.3 *Hspa1a* mRNA m6A Modification Mediates the Effects of FTO Regulation in MCAO/R and OGD/R Models

**FIGURE 4:**
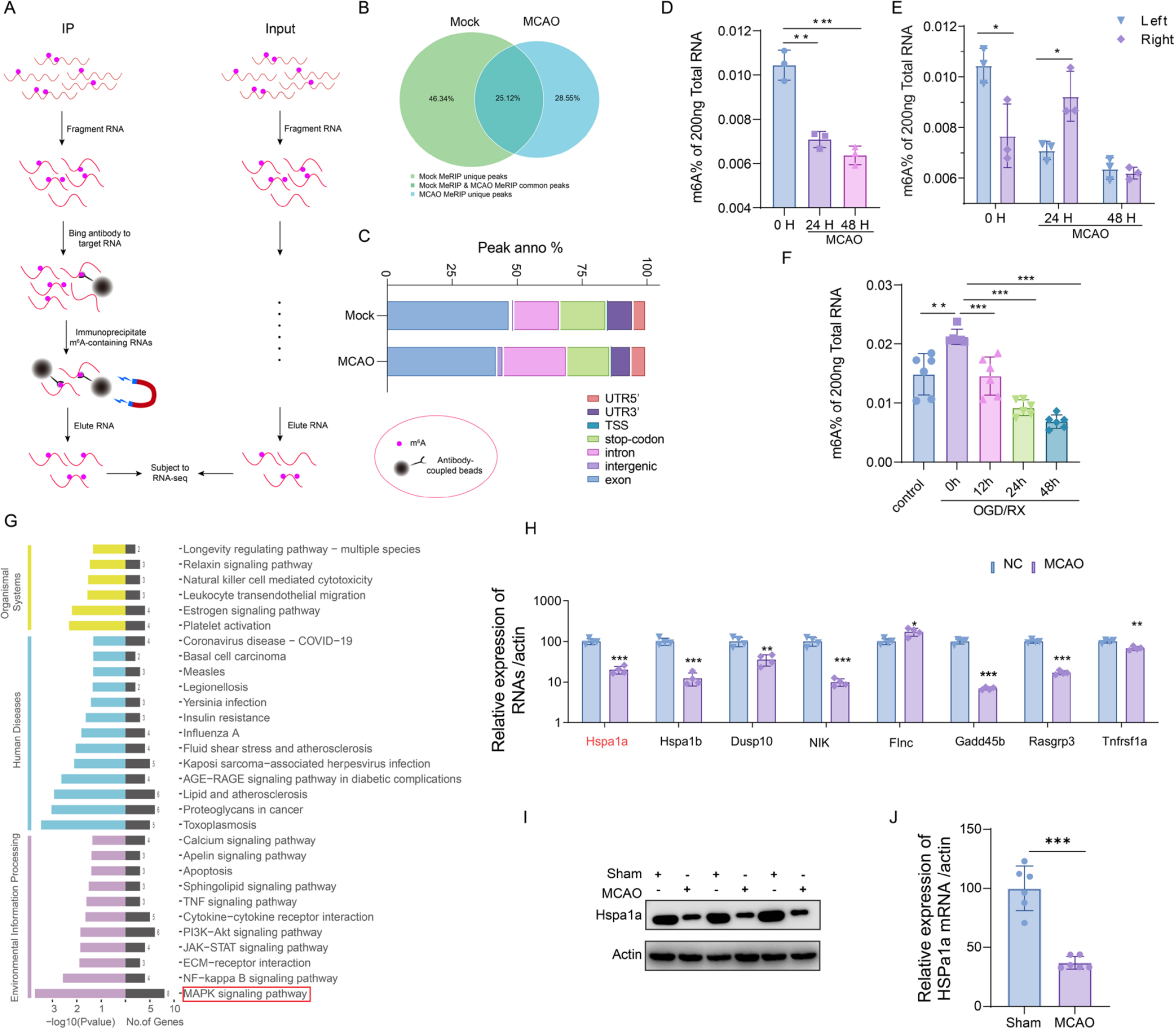
Mechanism of MCAO regulation by FTO. (A–C) MeRIP‐seq for changes in m6A mRNA levels before and after MCAO/R. (D–F) m6A mRNA levels decreased with time after. MCAO/R compared with the Sham control. *p < 0.05; **p < 0.01; ***p < 0.001. (G) KEGG pathway enrichment analysis of genes exhibiting significantly altered m6A modification levels. (H) qPCR for changes in genes involved in the MAPK pathway after MCAO/R. *p < 0.05; **p < 0.01; ***p < 0.001. (I, J) Western blotting and qPCR for changes in *Hspa1a* mRNA. ***p < 0.001.

Next, to investigate the molecular mechanisms by which FTO regulates the pathogenesis of CIRI, we employed MeRIP‐seq to detect changes in m6A methylation levels before and after MCAO/R. The results indicated that after CIRI developed, the overall m6A levels, particularly the offset exon and the 3′ untranslated region segment levels, decreased significantly (Figure 4A–C). To verify these results, we evaluated brain m6A levels over several time points after MCAO/R and noted that the decrease in overall m6A levels was more significant at the 48‐h timepoint than at the 24‐h timepoint (Figure 4D,E). These results were corroborated by the in vitro results in our OGD/R model (Figure 4F). Subsequently, MeRIP‐seq and KEGG pathway enrichment analyses revealed that the MAPK signaling pathway exhibited the most significant alterations following MCAO/R or OGD/R, particularly regarding the following genes: *Dusp10*, *Flnc*, *Gadd45b*, *HSPA1A*, *Hspa1b*, *Nlk*, *Rasgrp3*, and *Tnfrsf1a*. Of them, we selected *HSPA1A* as our gene of interest (Figure 4G). Next, we used Western blotting and qPCR to detect post‐MCAO/R changes in HSPA1A compared with a mock group (Figure 4H). The results confirmed that MCAO/R significantly reduced HSPA1A protein and mRNA expression (Figure 4I,J). Taken together, these results suggested that HSPA1A expression is associated with CIRI.

**FIGURE 5:**
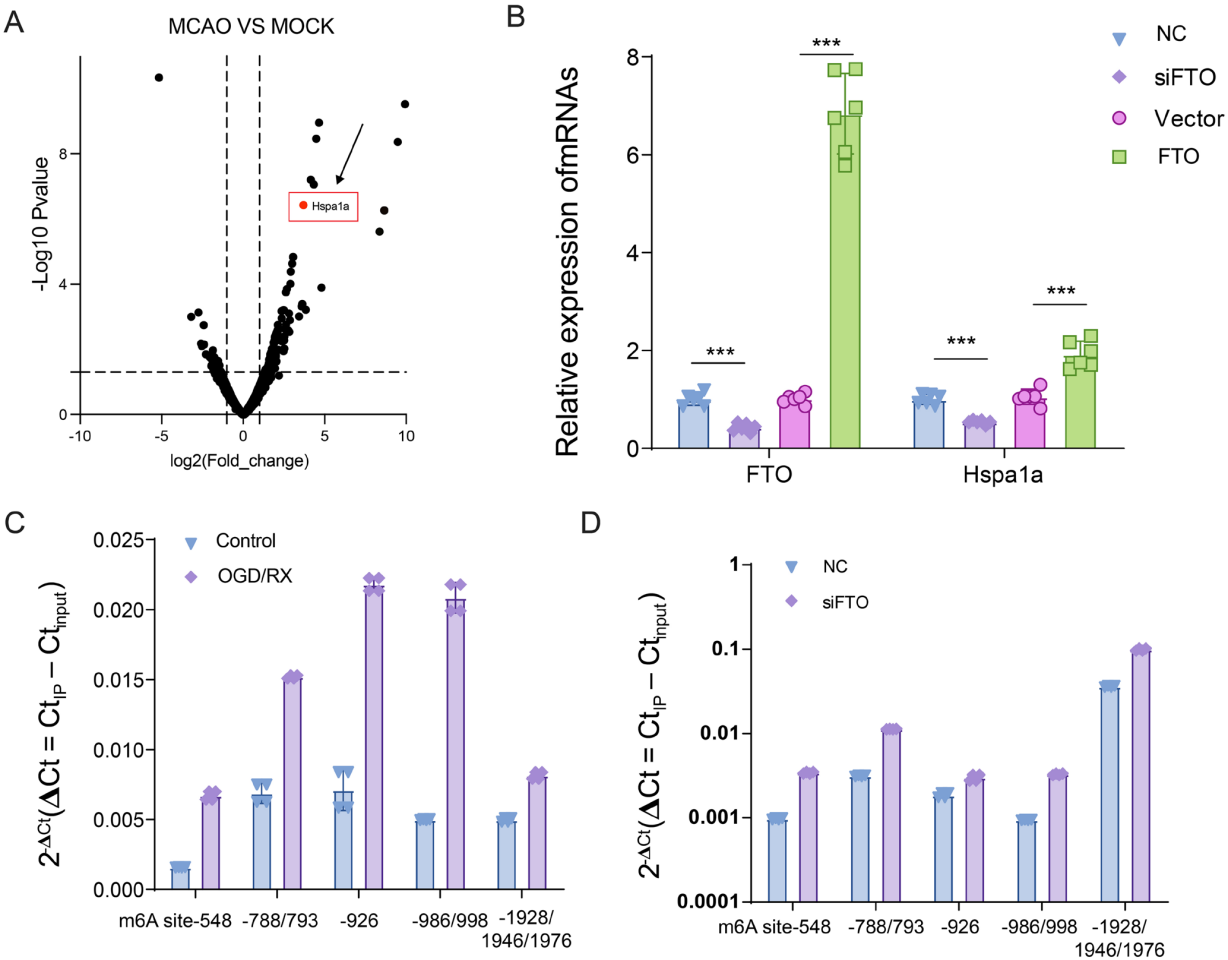
Regulation of MCAO by FTO through *Hspa1a* m6A RNA modification. (A) *Hspa1a* mRNA m6A modification in the MCAO/R model. (B) Changes in *Hspa1a* expression before and after FTO siRNA treatment and FTO over‐expression. ***p < 0.001. (C, D) Changes in m6A modification levels at different *Hspa1a* mRNA sites in the OGD/RX model without (C) or with (D) FTO siRNA treatment.

In the MCAO/R mice, the MeRIP‐seq results demonstrated that *Hspa1a* m6A modification was upregulated significantly (Figure 5A). Moreover, FTO knockdown led to a significant lowering of *Hspa1a* expression compared with the Sham control mice; in contrast, FTO overexpression led to a significant increase in *Hspa1a* expression (Figure 5B). We further noted an upregulation in m6A modification levels at different *Hspa1a* mRNA sites in the OGD/R model (Figure 5C). However, after FTO knockdown, these upregulated levels reduced significantly (Figure 5D). These results suggest that a decrease in FTO expression leads to elevated levels of m6A modification on *Hspa1a* mRNA, promoting its degradation and thereby alleviating CIRI.

### 3.4 FTO Regulates HSPA1A/NF‐κB/AQP4 Axis After MCAO/R and OGD/R

**FIGURE 6:**
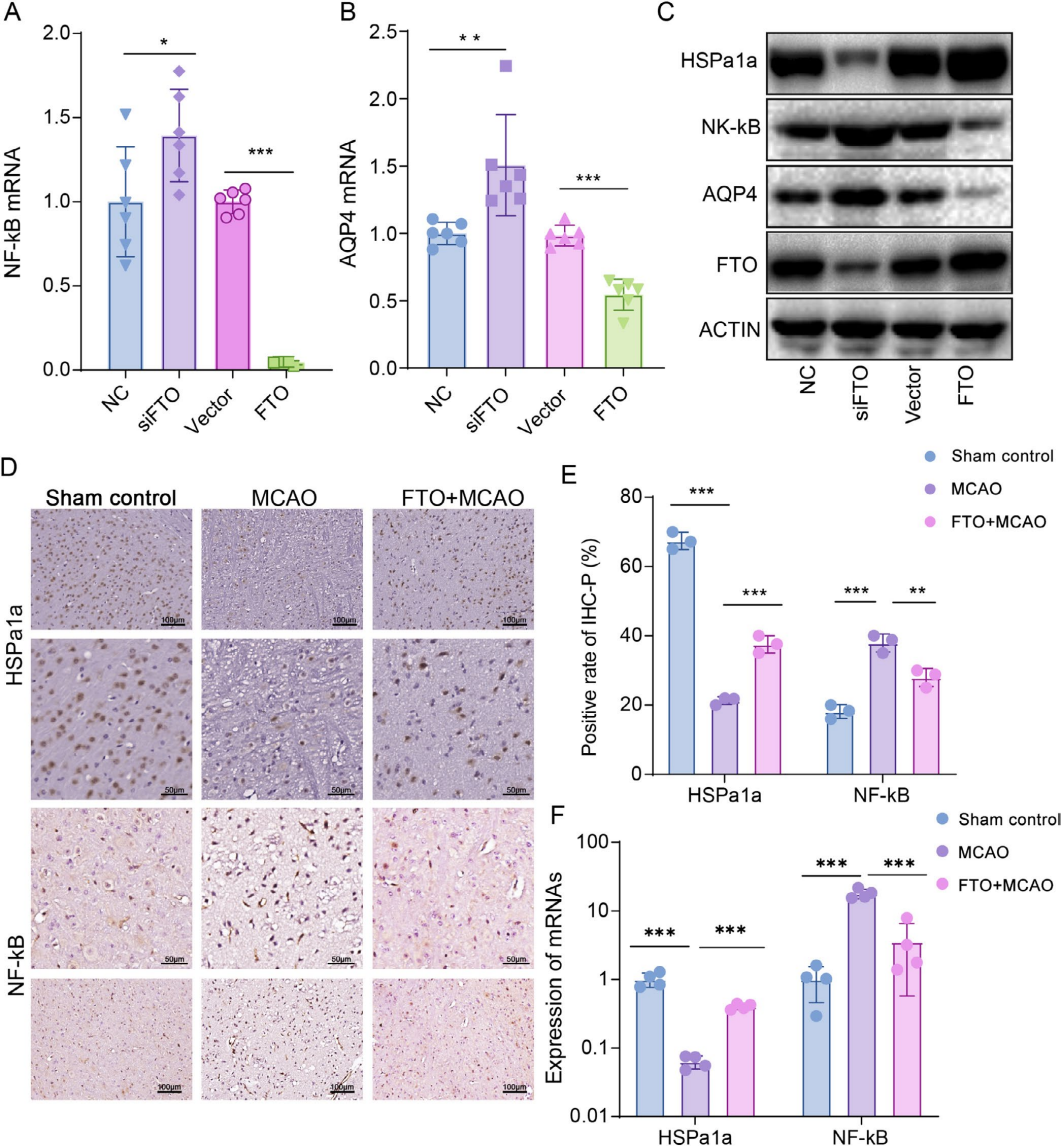
Participation of FTO in MCAO through *HSPA1A*/NF‐κB/AQP4 axis regulation. (A, B) qPCR results for changes in NF‐κB and AQP4 mRNA expression before and after FTO overexpression (A) or knockdown (B). *p < 0.05; **p < 0.01. (C) Western blot for changes in *HSPA1A*, NF‐κB, and AQP4 expression before and after FTO overexpression or knockdown. (D–F) Immunohistochemistry (D, E) and qPCR (F) for changes in *HSPA1A* and NF‐κB in mock, MCAO/R, and FTO + MCAO/R group rats. **p < 0.01; ***p < 0.001.

HSPA1A confers a protective effect against brain damage [30] and has been shown to inhibit NF‐κB activation [31]. NF‐κB also promotes inflammation by promoting AQP4 upregulation [32]. In addition, we previously confirmed that AQP4 plays a major role in CIRI development [22]. To investigate the role of FTO in CIRI development through the regulation of the *HSPA1A*‐NF‐κB‐AQP4 axis, we used qPCR to detect NF‐κB and AQP4 expression levels. The results demonstrated that NF‐κB and AQP4 expression decreased after FTO overexpression but increased after FTO knockdown (Figure 6A,B). To further validate this result, in MA‐C, FTO overexpression reduced NF‐κB and AQP4 expression but increased that of HSPA1A. However, with FTO knockdown, NF‐κB and AQP4 expression increased and HSPA1A expression decreased (Figure 6C). Finally, these results were corroborated by our immunohistochemistry results in the MCAO/R mice (Figure 6D,E).

**FIGURE 7:**
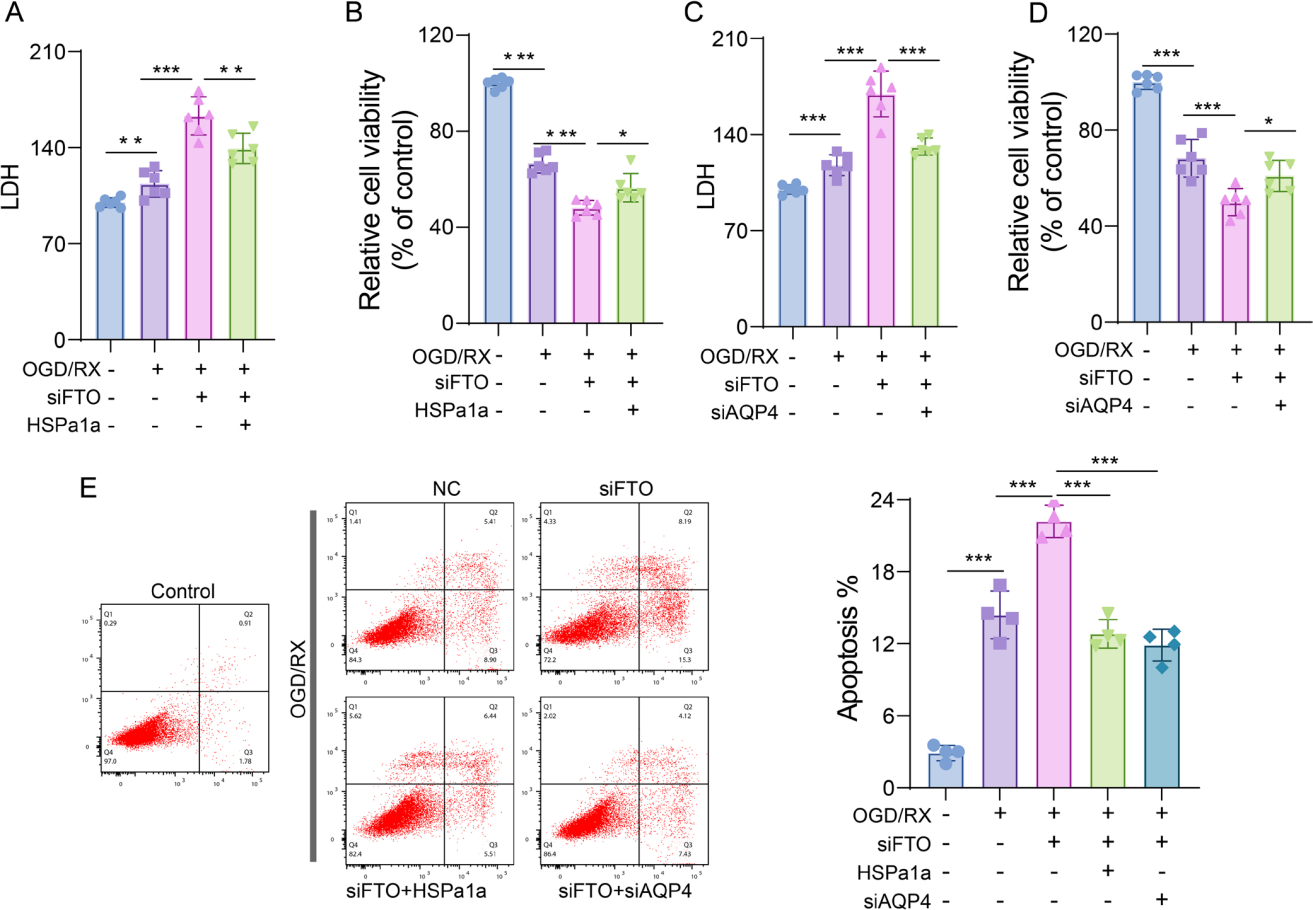
Protective effect of FTO through regulation of *Hspa1a* and AQP4 expression. (A, B) Astrocyte LDH levels (A) and viability (B) after FTO siRNA treatment, *Hspa1a* overexpression, or both. (C, D) Astrocyte LDH levels (C) and viability (D) after treatment with FTO siRNA, siAQP4, or both. E Flow cytometry results for apoptosis. *p < 0.05; **p < 0.01; ***p < 0.001.

To further verify that FTO regulates HSPA1A and AQP4 expression, we measured the viability and LDH levels in MA‐C. The increase in OGD/R damage due to FTO knockdown can be mitigated by HSPA1A overexpression (Figure 7A,B) and AQP4 knockdown (Figure 7C,D). Finally, these results were corroborated by the results of our flow cytometry analysis for apoptosis (Figure 7E).

## 4 Discussion

Following an ischemic stroke, the restoration of blood oxygen supply to the infarcted tissue (i.e., reperfusion) is essential. However, this process can paradoxically lead to CIRI [33], the pathogenesis of which involves highly complex mechanisms [34]. Consequently, exploring newer, more effective therapeutic strategies for CIRI is crucial. In the current study, we demonstrated that FTO suppresses AQP4 expression by regulating *Hspa1a* m6A modification, thereby alleviating CIRI (Graphical Abstract).

AQP4, a hydrophobic transmembrane protein primarily expressed in astrocytes, is closely linked to the prognosis of cerebral edema associated with CIRI [35, 36]. Furthermore, AQP4 represents a potentially important clinical target for the treatment of ischemic injury [23, 37, 38]. For instance, the upregulation of miR‐145 expression has been shown to reduce AQP4 levels and alleviate astrocyte damage under OGD/R conditions [22]. It has also been suggested that in CIRI, the m6A modification of lncRNAs exerts biological functions via lncRNA‐miRNA‐mRNA transcriptional networks [15]. For example, following CIRI in mice, m6A methylation levels were found to increase in 139 transcripts while decreasing in only 8 transcripts. These differentially m6A‐methylated mRNAs are primarily involved in inflammation, transcriptional regulation, and apoptosis [18]. Moreover, in renal ischemia/reperfusion injury, m6A modification was shown to increase PGC‐1α mRNA stability and protein expression, which further enhanced mitochondrial activity while simultaneously inducing oxidative stress [39]. However, there are relatively few studies regarding the regulation of AQP4 by m6A modification in the context of CIRI. Therefore, this study aimed to explore the underlying mechanisms and pathways governing this interaction.

m6A modification is the most prevalent post‐transcriptional epigenetic modification, and aberrant m6A RNA expression is frequently observed in the nervous system. Consequently, it is associated with various diseases, including depression, Alzheimer's disease, and CIRI [40]. Thus, the regulation of m6A modification appears to be critical in the progression of CIRI. Given that m6A modification is reversible [41], with FTO serving as its major regulator [42], and considering that FTO is normally highly expressed in brain tissue [43], we hypothesized that FTO plays a pivotal role in the pathology of CIRI. For instance, FTO expression is markedly downregulated in cortical neurons following cerebral ischemia, as well as in the peri‐infarct zone of myocardial tissue [44, 45]. Furthermore, it has been demonstrated that FTO overexpression suppresses FYN expression via m6A modulation, thereby inhibiting Drp1 activity and relieving CIRI [10]. Consistent with these findings, the present study demonstrates that upregulating FTO alleviates cerebral ischemia–reperfusion injury. We observed that FTO expression was significantly lower than that of other enzymes and declined progressively over time following MCAO/R. Similarly, in in vitro experiments, MA‐C subjected to OGD/R exhibited a significant reduction in FTO expression, corroborating the findings of Shao et al. [35]. Moreover, FTO overexpression effectively reduced apoptosis in OGD/R‐treated MA‐C, whereas transfection with FTO siRNA exacerbated apoptosis. These observations align with previous reports indicating that FTO overexpression promotes m6A demethylation and mitigates neuronal damage induced by cerebral ischemia [46]. Furthermore, compared to the MCAO/R group, intervention with FTO overexpression significantly reduced cerebral infarct volume and ameliorated neurological deficits. Collectively, these results suggest a protective effect of FTO against CIRI, which corroborates the findings of a previous study [47].

Heat shock proteins (HSPs) function as molecular chaperones, responsible for ensuring correct protein folding under physiological conditions and promoting the recovery of denatured proteins in cells exposed to stress [48]. Studies have demonstrated that HSPA1A can interfere with various cell death pathways, thereby exerting a protective effect in diverse injury models, including spinal cord injury, Parkinson's disease, and cardiac ischemia–reperfusion injury [16, 49]. Previous research has shown that the administration of HSPA1A inducers in rats with spinal cord injury can ameliorate tissue damage, promote neuronal survival, inhibit apoptosis, and facilitate the recovery of neurological function [50]. In the present study, we demonstrated that FTO alleviates CIRI by regulating the m6A modification of *Hspa1a*. Using MeRIP‐seq, we identified genes with significantly altered m6A modification levels before and after OGD/R. Subsequent KEGG pathway enrichment analysis revealed that the MAPK signaling pathway exhibited the most significant alterations, with *Hspa1a* showing the most marked decrease among the genes in this pathway. Moreover, the levels of m6A modification at various sites within *Hspa1a* mRNA were increased in the OGD/R model. Furthermore, transfection with FTO siRNA resulted in a significant decrease in HSPA1A protein expression in OGD/R‐treated MA‐C. In contrast, HSPA1A levels significantly increased following FTO overexpression. Therefore, a reduction in FTO expression leads to elevated m6A levels on *Hspa1a* mRNA, thereby promoting its degradation. In a related study on osteoblast injury, apoptosis induced by *Fto* knockout was found to be mitigated by *Hspa1a* overexpression [51]. Our study yielded similar findings; for instance, cell viability and LDH assays demonstrated that the exacerbation of OGD/R‐induced damage caused by FTO siRNA could be alleviated by HSPA1A overexpression. Consequently, the downregulation of FTO may aggravate brain damage by promoting *Hspa1a* m6A methylation, leading to a reduction in HSPA1A levels.

Decreased HSPA1A expression exacerbates CIRI, primarily because the reduction in HSPA1A levels diminishes its inhibitory effect on the NF‐κB proinflammatory axis, leading to NF‐κB activation [44]. Previous studies have demonstrated that suppressing the activated NF‐κB proinflammatory axis leads to reduced AQP4 expression and significantly attenuates astrocyte inflammation following OGD/R [52]. In this study, we found that FTO suppresses NF‐κB activity by upregulating HSPA1A, which in turn inhibits AQP4 expression, thereby alleviating CIRI. Compared with the OGD/R group, FTO overexpression resulted in increased HSPA1A expression, decreased NF‐κB and AQP4 levels, significantly enhanced cell viability, and reduced LDH release. Additionally, FTO overexpression combined with AQP4 knockdown significantly reduced cerebral infarct volume and ameliorated neurological deficits compared with the MCAO group. Furthermore, the exacerbation of OGD/R‐induced damage caused by FTO siRNA treatment was mitigated by HSPA1A overexpression or AQP4 knockdown. Collectively, these results suggest that FTO alleviates CIRI by modulating the HSPA1A/NF‐κB/AQP4 axis.

As emphasized by Balkaya et al., neurological severity scores are subjective and prone to false positives [53]. More critically, the authors note that due to spontaneous recovery typically occurring within 1–2 weeks, the modified neurological severity score (mNSS) “tends not to be useful for long‐term studies.” This rapid spontaneous recovery aligns with the temporal dynamics relevant to our experimental timeline. Consequently, relying solely on such scores could obscure genuine treatment effects or underlying pathophysiological changes that manifest as subtle functional impairments. Therefore, while the Bederson score served a valid purpose in our study for confirming the successful establishment of the MCAO model at an early time point, we acknowledge its limitations as a primary outcome measure for evaluating the nuanced effects of FTO manipulation on functional recovery. Consistent with the recommendations of Balkaya et al. future investigations into therapeutic targets such as FTO would benefit from incorporating more sensitive, objective, and quantitative behavioral assessments, such as the adhesive removal test, cylinder test, or skilled reaching tasks, which are capable of detecting long‐term sensorimotor deficits and differentiating true functional recovery from compensatory mechanisms.

We acknowledge that laser Doppler flowmetry (LDF) is considered the gold standard for real‐time monitoring of cerebral blood flow, and we recognize the absence of LDF data as a limitation of the current study. However, to ensure the reliability of MCAO induction in the absence of LDF, we implemented strict functional and behavioral inclusion criteria. Specifically, proper occlusion was confirmed post‐operatively using the Bederson score. As detailed in the Methods section, all mice were assessed at 24 h post‐surgery; only animals exhibiting clear neurological deficits (Bederson score ≥ 1) were included in the analysis, while those displaying no deficits (score = 0) were considered model failures and were strictly excluded. This functional screening ensures that all animals included in the study, including those represented in the TTC staining in Figure 3B, definitively suffered ischemic injury. Consequently, the reduced infarct sizes observed in the treatment groups reflect the protective efficacy of the intervention rather than insufficient occlusion.

## 5 Conclusion

In conclusion, the present study demonstrates that FTO levels are significantly downregulated following CIRI. This reduction leads to increased m6A methylation of *Hspa1a* mRNA and promotes its degradation. The consequent loss of HSPA1A results in the upregulation of NF‐κB and AQP4 expression, thereby exacerbating CIRI. These findings identify the FTO/HSPA1A/NF‐κB/AQP4 axis as a potential therapeutic target and provide a theoretical basis for the development of novel treatment strategies for ischemic stroke.

## Author Contributions

Wei Chen and Hongwei Wang designed the study and collected data. Luyuan Cao, Xiaoxiao Zheng, Li Zheng, Jiahua Lan, and Cheng Jiang did the experiments. Qing Wang and Ting Guan analyzed the data. Wei Chen wrote the manuscript. Wei Chen and Hongwei Wang reviewed and edited the manuscript. All authors read and approved the final manuscript.

## Funding

This work was supported by the National Natural Science Foundation of China (grant numbers 82174132 and 82374293), the Natural Science Foundation of Guangdong Province (grant number 2020A151501287), the General Project of Science and Technology Innovation Commission of Shenzhen (grant number JCYJ20190808103401655), and the Medical Health Science and Technology Project of Zhejiang Province (grant number 2025ZR085).

## Ethics Statement

This study was approved by the Experimental Animal Welfare Ethics Committee of the Zhejiang Academy of Traditional Chinese Medicine (2024‐062).

## Conflicts of Interest

The authors declare no conflicts of interest.

## Data Availability

The data that support the findings of this study are available from the corresponding author upon reasonable request.
